# Special Issue “Molecular Precision Medicine: Unraveling Novel Mechanisms and Delivery Strategies in Complex Diseases”

**DOI:** 10.3390/ijms26157439

**Published:** 2025-08-01

**Authors:** Nuno Vale

**Affiliations:** 1 PerMed Research Group, RISE-Health, Faculty of Medicine, University of Porto, Alameda Professor Hernâni Monteiro, 4200-319 Porto, Portugal; nunovale@med.up.pt; 2 Laboratory of Personalized Medicine, Department of Community Medicine, Health Information and Decision (MEDCIDS), Faculty of Medicine, University of Porto, Rua Doutor Plácido da Costa, 4200-450 Porto, Portugal; 3 RISE-Health, Department of Community Medicine, Health Information and Decision (MEDCIDS), Faculty of Medicine, University of Porto, Rua Doutor Plácido da Costa, 4200-450 Porto, Portugal

In recent years, advancements in molecular precision therapeutics have profoundly reshaped the understanding and treatment of complex diseases. These approaches integrate multilayered data, from genomics and proteomics to clinical and environmental information, to identify highly specific therapeutic targets and design tailored interventions. This Special Issue highlights these emerging strategies, with a particular focus on drug repurposing and innovative delivery systems that aim to enhance therapeutic efficacy and improve clinical outcomes. Among the most promising strategies in this domain is drug repurposing, which has gained increasing attention, particularly in oncology. By leveraging already-approved compounds with well-established safety and pharmacokinetic profiles, drug repurposing accelerates the discovery of new therapeutic applications while reducing the costs and timelines associated with traditional drug development. Enabled by technologies such as high-throughput screening, computational modeling, molecular docking, machine learning, and patient-derived organoids, repurposing has become a data-driven and integrative discipline within precision medicine. Beyond monotherapy, repurposed drugs have shown potential in combination therapies, radiosensitization, side effect management, and recurrence prevention. Examples include curcumin, metformin, sertraline, fluphenazine, efavirenz, and levosimendan, all of which have demonstrated activity on key cancer-related molecular pathways such as Akt, Wnt, TNF-MAP4K4-JNK, and fatty acid metabolism, highlighting the polygenic and multifactorial nature of many diseases.

Despite their promise, repurposing efforts still face regulatory and economic challenges, particularly concerning intellectual property protection and funding for late-phase clinical trials. Academic–industry collaboration, supported by public policy incentives, will be essential to unlock the full potential of these therapies. Over the past few decades, the field of oncology has made significant strides in treatment, including improvements in surgery, radiation therapy, and chemotherapy. However, they frequently cause the patient to have significant side effects, and they eventually cause tumor cells to become resistant to the drugs. Drug repurposing, a promising method that involves finding new therapeutic applications for already-approved drugs, has attracted a lot of interest. This strategy makes use of the proven pharmacokinetics and safety characteristics of these drugs, which can accelerate the creation of novel cancer treatments and lower related expenses [1]. The ability of drug repurposing to avoid many of the time-consuming and expensive stages of standard drug development makes it particularly attractive in the field of cancer. Researchers can concentrate on assessing the effectiveness of drugs for cancer treatment by repurposing them after they have already received approval for other uses.

Drug repurposing techniques come in a variety of forms and typically consist of three steps. The process of generating hypotheses first determines the main targets of the disease. Preclinical testing then assesses the drug’s effectiveness in vivo (using animal models) and in vitro (using cell cultures). Ultimately, drugs that prove beneficial move on to phase II clinical trials, which typically begin if there is enough data available beforehand. The process of identifying repurposed drugs involves both computational and experimental methods. Large drug libraries are screened using experimental approaches on disease models, such as organoids produced from stem cells, which replicate the structure and functionality of human cancer tissues. As a result, scientists may evaluate a drug’s potential without requiring specific molecular targets. Meanwhile, computational techniques like data mining, pathway analysis, molecular docking, and machine learning are used to choose promising candidates and forecast drug–disease interactions [2].

Repurposed drugs are important to cancer treatment because they may be used for monotherapy, combination therapy, managing side effects, and sensitizing the body to radiation and chemotherapy. Drugs with particular modes of action are used in monotherapy to stop the growth of tumors or kill cancer cells. In the case of glioma, for example, repurposed drugs are explored to target cancer stem cells [3]. Repurposed drugs may also be used as prophylactics in at-risk groups, to stop metastases, or to lower the chance of cancer recurrence. Curcumin, for instance, is being researched for low-risk prostate cancer [4], while metformin is being researched to prevent breast cancer in high-risk patients and the recurrence of endometrial cancer [5].

The problem of cancer stem cells causing metastasis and recurrence complicates cancer therapy. As knowledge of cancer biology expands, so does the significance of combination therapy considering the intricate nature of tumors, their microenvironment, and many signaling pathways. When treating tumors, a multi-target strategy works better than a single-target strategy. Combination treatments raise the expense of therapy. Nevertheless, these tactics are more accessible and economical when they use repurposed drugs [6]. Combinations of drugs with new purposes, such as nelfinavir, can also improve the effectiveness of conventional chemoradiotherapy. Combination drugs’ synergistic effects enable lower therapeutic dosages without sacrificing cytotoxicity against cancer cells or harming healthy cells. Furthermore, repurposed drugs may have “off-target” effects that provide unexpected anticancer advantages. One example of this is the way that etomoxir affects the growth of cancer cells by means of fatty acid pathways [7].

Implementing drug repurposing still presents several obstacles, mainly related to issues with business and intellectual property. The financial benefits of market exclusivity—typically attained through regulatory exclusivity and patent protection—are crucial to pharmaceutical businesses. Repurposed drugs, however, are less profitable as their active components cannot be covered by new patents because they are already well-known products. Although “use” patents can shield novel indications, their enforcement is more difficult and costly, frequently resulting in the off-label prescription of less expensive generic alternatives and reducing potential earnings. Since phase II and III trial costs are a significant obstacle, policy reforms have been proposed to support medication repurposing, including tax incentives and financing for clinical studies. Repurposing generic drugs is mostly dependent on academia and independent research, with partnerships between academia and the pharmaceutical sector providing a hopeful future [8].

In drug repurposing clinical trials, clinical equipoise is also essential to maintain a balance between routine care and exploring novel treatments. Although a drug’s toxicity and side effects can help evaluate how safe it is to repurpose, studies need to determine whether the drug works well for its new purpose. For the new indication, a greater dose could be required for efficacy, and incorrect dosing—rather than the medication itself—may be the cause of failure. A research project must also be financially feasible and have sufficient statistical power. Researchers must make sure that delaying necessary treatments is appropriate by comparing the repurposed drug to the standard of care. Patient outcomes might be jeopardized in the absence of proof of the repurposed drug’s effectiveness. Therefore, in order to guarantee the validity of drug repurposing research, ethical issues are crucial [9].

Because cancer tumors are polygenic, combination therapy presents additional challenges in the treatment of cancer. Trials involving combination drugs are more intricate and costly than those involving monotherapy, and several promising treatments have failed in phase II studies because they do not exhibit single-agent activity. To address these issues, more trustworthy preclinical research and high-throughput screening techniques are required to evaluate the effectiveness of drug combinations prior to the expensive and time-consuming requirement for clinical trials [10].

Several notable examples demonstrate the success of drug repurposing in oncology and note the potential of repurposed drugs to provide new therapeutic options for cancer patients. This Special Issue highlights some examples of repurposed drugs for cancer treatment, with five articles on the topic present in this issue. The antipsychotic drug fluphenazine is used for long-term neuroleptic therapy, such as schizophrenia and other psychotic disorders. It has been demonstrated to have cytotoxic effects in several cancer cell types, like lung and breast, and is often associated with alterations in the proteins associated with tumor growth such as P-glycoprotein and ABCB1, as well as in cancer pathways like the Akt and Wnt signaling pathways, something that is explored in one of the reviews in this Special Issue [11]. Another central nervous system drug with cancer-repurposing potential depicted in this issue is sertraline, an antidepressant of the class of selective serotonin reuptake inhibitors. In vitro and in vivo studies demonstrated the anticancer effect of sertraline on various cancer cell models, with this drug targeting P-glycoprotein and cancer pathways like TNF-MAP4K4-JNK, with ultimate effects in cell cycle arrest, DNA fragmentation, and apoptosis [12].

Another class of drugs often explored for repurposing is antivirals, and among them, efavirenz is one of the more promising. This is a first-generation non-nucleoside reverse transcriptase inhibitor that is used in combination for the treatment of HIV infection. Ample research has been made on efavirenz repurposing in several cancers, like colorectal and pancreatic, with fatty acid metabolism being one of the main pathways attributed to the anticancer effect of efavirenz [13]. The last of the reviews present in this Special Issue focuses on levosimendan, a calcium sensitizer previously approved for heart failure. This drug demonstrated several anticancer properties, such as reactive oxygen species reduction and cancer cell sensitization to radiation, as well as synergistic effects with antineoplastic drugs such as 5-fluorouracil. This can help in the reduction of anticancer drug doses, with the consequent decrease in side effects associated with these drugs in patients [14].

This Special Issue aims to foster an interdisciplinary and translational perspective in molecular precision medicine, with emphasis on the discovery of novel therapeutic molecules, molecular biomarkers, and innovative delivery systems (e.g., nanotechnology, biopolymers, and targeted vectors). We invite readers to explore the featured contributions, which showcase how synthetic biology, bioinformatics, and translational pharmacology are converging to redefine the therapeutic landscape.
